# Fierce poison to others: the phenomenon of bacterial dependence on antibiotics

**DOI:** 10.1186/s12929-023-00963-x

**Published:** 2023-08-14

**Authors:** Claudia C. Paredes-Amaya, María Teresa Ulloa, Víctor Antonio García-Angulo

**Affiliations:** 1 Microbiology Department, Escuela de Ciencias Básicas, Facultad de Salud, Universidad del Valle, Cali, Colombia; 2 Microbiology and Micology Program, Facultad de Medicina, Instituto de Ciencias Biomédicas, Universidad de Chile, Independencia 1027, Independencia, RM, Santiago, Chile; 3 Vertebral I+D+i - Corporation for Assistance for Burned Children (Coaniquem), Santiago, Chile

**Keywords:** Antibiotic dependence, Antimicrobial resistance, Vancomycin, Linezolid, Colistin

## Abstract

Beyond the development of resistance, the effects of antibiotics on bacteria and microbial communities are complex and far from exhaustively studied. In the context of the current global antimicrobial resistance crisis, understanding the adaptive and physiological responses of bacteria to antimicrobials is of paramount importance along with the development of new therapies. Bacterial dependence on antibiotics is a phenomenon in which antimicrobials instead of eliminating the pathogens actually provide a boost for their growth. This trait comprises an extreme example of the complexities of responses elicited by microorganisms to these drugs. This compelling evolutionary trait was readily described along with the first wave of antibiotics use and dependence to various antimicrobials has been reported. Nevertheless, current molecular characterizations have been focused on dependence on vancomycin, linezolid and colistin, three critically important antibiotics frequently used as last resource therapy for multi resistant pathogens. Outstanding advances have been made in understanding the molecular basis for the dependence to vancomycin, including specific mutations involved. Regarding linezolid and colistin, the general physiological components affected by the dependence, namely ribosomes and membrane function respectively, have been established. Nonetheless the implications of antibiotic dependence in clinically relevant features, such as virulence, epidemics, relationship with development of resistance, diagnostics and therapy effectiveness require clarification. This review presents a brief introduction of the phenomenon of bacterial dependence to antibiotics and a summary on early and current research concerning the basis for this trait. Furthermore, the available information on the effect of dependence in key clinical aspects is discussed. The studies performed so far underline the need to fully disclose the biological and clinical significance of this trait in pathogens to successfully assess its role in resistance and to design adjusted therapies.

## Introduction

It only took a few years after the first introduction of antibiotics in clinics for antibiotic-resistant bacterial strains to appear [1]. To date, resistances to all antibiotics used to treat human infections have been identified [2]. Given its paramount significance for global public health, the occurrence of antibiotic resistance is a very active research area and novel mechanisms are being constantly identified. Bacteria may develop antibiotic resistance by the acquisition of chromosomal mutations and by horizontal gene transfer of resistance determinants. This endows bacteria with different ways to cope with antimicrobials such as modification of targets, acquisition of enzymes able to modify the antibiotic or target bypass by the gain of alternative pathways that replace the original native antibiotic target, among other genetic-based mechanisms. Nonetheless, resistance can also be the result of intrinsic, non-inheritable characteristics that diminish the effect of or the exposure to the antibiotic. This is known as phenotypic resistance and may be conferred by diminished bacterial metabolic activity, the development of the persistence status by a subset of the population, changes in antibiotic permeability or extrusion, surface composition modification, biofilm formation and increase in the surface area by overproduction of outer membrane vesicles, among other mechanisms [2–4].

After decades of investigation on this subject, it is acknowledged that many antibiotics have more bacterial targets than originally thought [5], and beyond the development of resistance, the effects of antibiotics in bacterial physiology and evolution are complex. Frequently, the acquisition of antibiotics resistance by bacteria is accompanied by different evolutionary trade-offs. A common example of this is collateral sensitivity, in which the generation of resistance against one antibiotic leads to an increased susceptibility to another drug. Another important trade-off is the fitness cost, on which the resistant bacteria is less fitted to grow in the absence of antibiotics than the parental susceptible bacteria. Integral knowledge regarding these and other effects of antimicrobials on bacterial physiology can be exploited to design better strategies to combat the antibiotic resistance crisis [6].

**Fig. 1:**
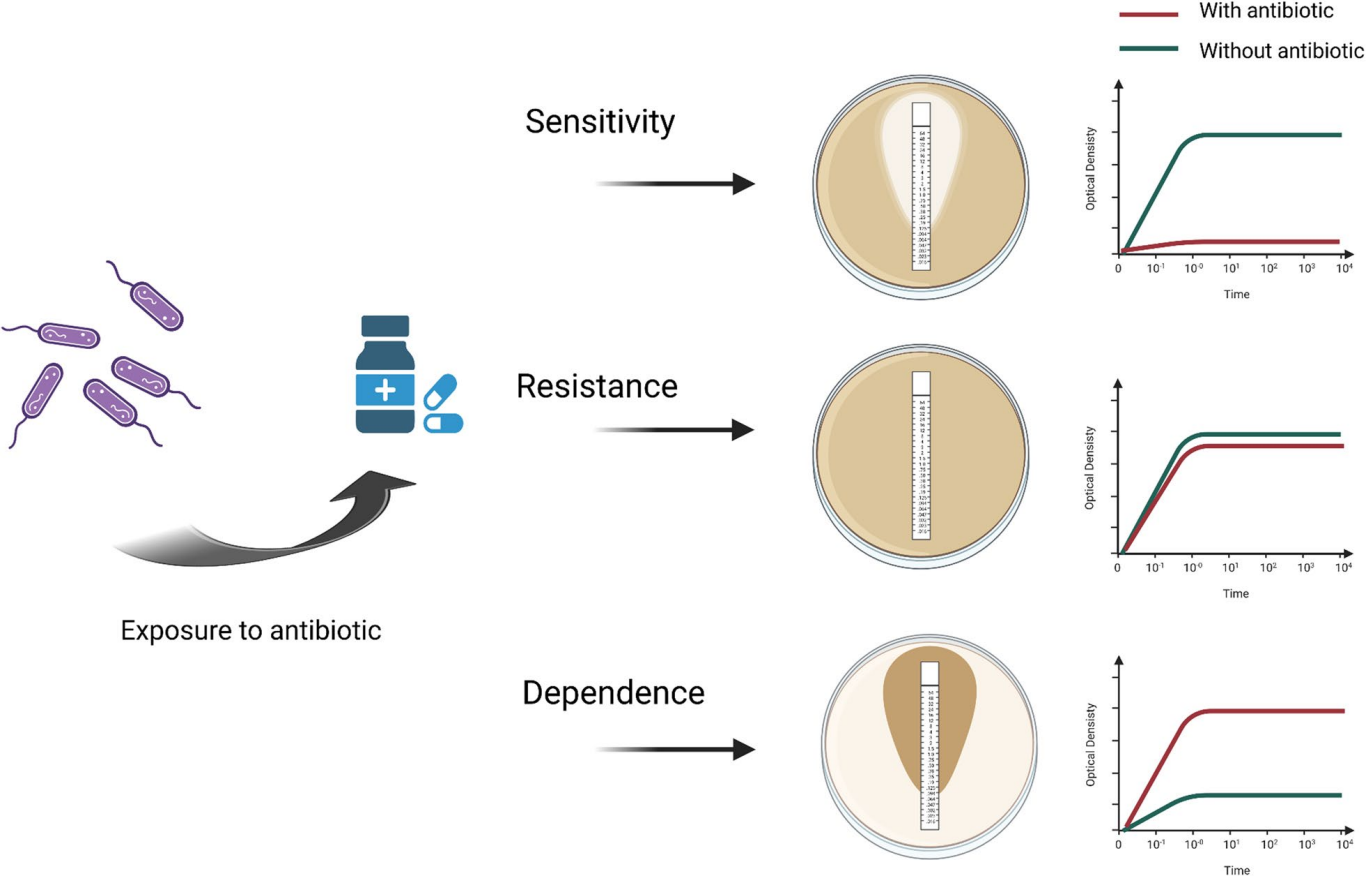
Antibiotic sensitivity, resistance and dependence. Different bacterial responses to antibiotic exposure are represented in E-test assays and growth in liquid media. A drug-sensitive bacteria is inhibited by the antibiotic gradient emanating from the E-test strip in a concentration-dependent manner (growth is represented by a brown lawn). In liquid medium, the addition of antibiotics avoids the growth of the bacteria. An antibiotic-resistant bacteria is able to grow adjacent to the E-tes strip and its growth is barely affected by the presence of antibiotics in liquid medium. The growth of dependent bacteria is only observed in the area where the antibiotic has diffused from the E-test strip. In liquid media, its growth curve is significantly improved by the addition of the antibiotic

An intriguing and increasingly recognized trait developed by bacteria is antibiotic dependence. This is defined as the requirement of an antibiotic by a bacterium to grow, or to a high improvement in bacterial growth provided by an antibiotic [7–9]. Facing an antibiotic challenge, typical naive bacteria may display sensitivity, meaning the drug is able to kill the bacteria. Alternatively, resistant bacteria may remain unaffected or exhibit only little adverse effects in growth (Fig. 1). By contrast, dependent bacteria grow only in the presence of the antibiotic, or a strain with defective growth is significantly improved by the antibiotic. In Fig. 1, this is schematized by the gradient of microbial growth lining the increasing drug concentration diffusing from the antibiotic strip in an E-test assay, or by an acceleration of the growth rate during a growth kinetics curve in a liquid medium with antibiotic. In many cases, clinical development of dependence is preceded by large courses or antibiotics. The development of dependence has been described as the ultimate step in antibiotic resistance [10], although it is not yet clear whether this trait represents a fitness cost associated with resistance acquisition or a compelling case of adaptive evolution. In a few cases, the molecular basis for the dependence have been established, with mutations responsible for the trait clearly identified, while in most instances antibiotics dependence remains an empirical observation. The insights obtained about the mechanisms through which antibiotics improve growth in dependent strains indicate that they may be required for the induction of the expression of key survival genes originally acquired as a resistance mechanism, or for functional restoration of the bacterial components composing their primary targets like ribosomes or the cell membrane.

Bacterial antibiotic dependence is a long-time known trait. To the best of our knowledge, the first report of clinical dependence tracks back to 1947, when Hall and Spink reported a streptomycin-resistant *Brucella* strain isolated from a brucellosis patient who received a 31 day intramuscular streptomycin treatment. The growth of this isolate was enhanced by streptomycin [11]. Soon, others reported the rise of streptomycin-dependent variants of many different bacteria when submitted to in vitro resistance development [12–14]. Since then, several studies have documented antibiotic-dependence in pathogenic bacteria in naturally occurring clinical isolates as well as in strains derived from in vitro resistance evolution studies. Besides streptomycin, examples of antibiotics that generate dependence include terramycin, aureomycin and chloromycetin [15], chloramphenicol [16], rifampicin [17, 18], erythromycin [19], penicillin and ceftriaxone [20], sulfamethoxazole [21], vancomycin [7], linezolid [22], and polymyxin B and colistin [23, 24], among others. Moreover, evolution towards dependence to initially detrimental drugs is a trait distributed across different kinds of pathogens. For example, the emergence of a human immunodeficiency virus type 1 mutant dependent on the fusion inhibitor T20 drug has been reported [25] and a variant of the pathogenic yeast *Candida albicans* requires high concentrations of the caspofungin fungicidal to proliferate in a pattern known as “paradoxical growth” [26].

Evolution from antibiotic sensitivity to resistance and dependence is a fascinating biological phenomenon illustrating the extraordinary adaptability capacities of bacteria and the complexity of the effects of antibiotics in microbial ecology. Antibiotic dependence in human pathogens has deep implications in diagnosis and clinical therapy that have been only scarcely investigated. Such implications include the extent to which dependent pathogens escape detection because of the lack of antibiotics in the primary isolation cultures and the ramifications of the putative role of antibiotic therapies in pathogen fitness when infections are caused by dependent bacteria. Some of the basis and implications of antibiotic dependence in bacterial pathogens started to be studied soon after its unveiling in the late 1940s. Then, some remarkable advances were made, particularly on the characterization of streptomycin-dependent strains. In recent years, although dependence on numerous antibiotics has been observed, further characterization has been focused on vancomycin, linezolid and colistin. These three antibiotics are of special interest as they are often used as last-resource therapy against several multi-resistant bacteria. This review summarizes knowledge on the observations and characterization of antibiotic dependence of bacterial pathogens to antibiotics. It presents a brief description of the main pioneering studies in the subject and deepens into more recent advances of the molecular basis and clinical implications of this trait with a special emphasis in the clinically relevant vancomycin, linezolid and colistin.

## Early research on antibiotic dependence

Bacterial antibiotic dependence is a trait that was observed even for drugs used in the first wave of the clinical employment of antibiotics. A biphasic mode of action for penicillin, on which sub-inhibitory concentrations seemed to increase the growth of sensitive *Staphylococcus aureus* was readily described in 1945 [27]. However, clinically relevant antibiotic dependence was first documented for streptomycin. This was the second therapeutically useful antibiotic right after penicillin and the first successful cure for tuberculosis and other infections caused by Gram negative pathogens [28]. After the first report of dependence on streptomycin in 1947 in a *Brucella* clinical strain, other pathogens, including *Escherichia coli, Pseudomonas aeruginosa* and *Mycobacterium* that were boosted by streptomycin during in vitro resistance development experiments were reported the same year [12, 13, 29] or within the following years [14, 30]. In addition, the growth of other nonpathogenic bacteria including *Bacillus subtilis* [12] (Kushnick, 1947) and *Bacillus megaterium* were also noticed to be stimulated by low concentrations of streptomycin or penicillin [31]. In the following decades, examples of other antibiotics generating dependence were described, including terramycin, aureomycin and chloromycetin [15], chloramphenicol [16], rifampicin [17, 32], erythromycin [33], spectinomycin [34] and kasugamycin [35].

In this early stage of identification of the antibiotic dependence phenotype, some further characterization of the trait was performed. In most cases, dependence was shown to involve mutations in ribosomal proteins or ribosome-interacting proteins with some involvement of transcription related factors [33, 35–37]. Also, it was noticed that some strains dependent on a ribosomal-impairing antibiotic may be trans-relieved by other ribosomal-interacting antibiotics [19, 32]. Probably because of its therapeutic importance, the streptomycin dependence was significantly better characterized. Streptomycin is an aminoglycoside antibiotic that impairs translation by binding the 30S ribosomal subunit and inducing a distortion of the 16S ribosomal RNA, which interferes with codon recognition [38]. Initial investigations showed that streptomycin dependence in *E. coli* sets because of mutations in the ribosomal protein S12 from the 30S subunit [39] and that mutations in the S10 ribosomal protein revert the dependence phenotype [40]. Later, it was demonstrated that ribosomes from dependent mutants have a highly diminished translation rate and are over-accurate due to an enhanced proofreading activity. In these strains, streptomycin stimulates growth by increasing translation efficiency through the induction of proofreading loss [41, 42]. Notably, owed to these characteristics, the ribosomes of streptomycin-dependent strains were thoroughly used as tools for pioneering studies assessing different translation-related processes such as the role of translation rate in mutation [43], the mechanism of action of suppressor tRNAs [44], programed translational frameshift [45, 46] and allelic recombination processes [47]. Moreover, the advantageous features of ribosomes derived from streptomycin-dependent strains were employed in modern works to analyze the activity of the SARS-CoV-2 RNA-dependent RNA polymerase [48] and to measure direct translation kinetics within living cells at codon resolution [49]. Despite this, many further aspects of the molecular basis and the biological and clinical significance of the dependence on the group of antibiotics that were firstly acknowledged still need to be clarified.

## Bacterial dependence to last resort antibiotics

Most recent research has been focused on antibiotics being intensively used to treat multidrug resistant pathogens. Vancomycin, linezolid and colistin are known to generate strains of antibiotic dependent pathogens. Vancomycin and linezolid are recurrently used as last-resource therapy against some Gram positive pathogens [50]. Likewise, colistin is frequently used as a last line antibiotic against carbapenem-resistant Gram negative bacteria [51]. These three antibiotics have been listed as part of the “Critically Important Antimicrobials for Human Medicine” by the World Health Organization (WHO CIA list), meaning they are often the sole available therapy to treat serious bacterial human infections caused by strains prone to acquire antibiotic resistance (https://www.who.int/publications/i/item/9789241515528, accessed on 3/03/2023). Moreover, in the WHO Access, Watch, Reserve (AwaRe) classification of antibiotics to guide antibiotic stewardship systems, vancomycin has been assigned to the Watch group, while linezolid and colistin have been classified into the Reserve. The Reserve group comprises antibiotics that should be reserved for the treatment of confirmed or suspected infections due to multidrug-resistant pathogens. (https://www.who.int/publications/i/item/WHO-MHP-HPS-EML-2021.02, accessed on 3/03/2023). Thus, the WHO categorization of these three drugs reflects the importance they pose to current antimicrobial therapies in the context of the current global antibiotic resistance challenge. Given the role of these drugs in public health, a deep understanding of the physiology of this trait is paramount.

## Bacterial dependence on vancomycin

Vancomycin is a tricyclic glycopeptide used against the Gram positive *S. aureus*, *Staphylococcus epidermidis*, *Streptococcus pyogenes*, *Streptococcus pneumoniae*, *Streptococcus viridans,* Enterococci and some species of *Bacillus*, *Actinomyces*, *Clostridium* and *Corynebacterium* [52]. This antibiotic interferes with the synthesis of the cell wall by impeding peptidoglycan maturation, affecting cell envelope and causing bacterial death. Specifically, vancomycin binds the _D_-Ala-_D_-Ala terminal moiety of the peptide chain component of the structural subunit, impeding normal peptidoglycan layer maturation [53].

The acquisition of vancomycin resistance by *Enterococcus* species and *S. aureus* is of particular concern in clinical practice [53–55]. The mechanism of vancomycin resistance involves the degradation of the _D_-Ala-_D_-Ala natural substrate for peptidoglycan biosynthesis and the synthesis of the alternative precursors _D_-Ala-_D_-Lac or _D_-Ala-_D_-Ser which are not recognized by the antibiotic. Two different genetic cassettes mediate this. The replacement of _D_-Ala-_D_-Ala by _D_-Ala-_D_-Lac is allowed by a cluster of five genes, the *vanHAX* operon encoding the VanH, VanA and VanX enzymes, and the regulatory *vanRS* operon [53, 55]. VanH, VanA and VanX cleave the native _D_-Ala-_D_-Ala and synthesize the alternative _D_-Ala-_D_-lac substrate, while the *vanRS* locus codes for a two-component system (TCS) for signal transduction [56]. Vancomycin in the environment is sensed by VanS, a membranal histidine kinase sensor that activates the VanR transcriptional regulator which then becomes able to interact with DNA to activate transcription of the *vanHAX* promoter making the bacteria able to cope with the antibiotic [56–58]. This system is denominated the VanA-type of resistance and variations of this system make use of different *vanA* homologous forms in the operon. This resistance mechanism also includes the *vanY* and *vanZ* accessory genes [59]. A second type of vancomycin resistance, which synthesizes peptidoglycan precursors with the a _D_-Ala-_D_-Ser motif, was originally called VanC type resistance. This was due to the presence of the VanC _D_-Ala-_D_-Ser ligase that is present in the set of genes responsible for this resistance in *Enterococcus* species [60, 61]. Later, the VanE, VanG, VanL and VanN types of _D_-Ala-_D_-Ser ligases were found in vancomycin resistance cassettes from other bacterial species [62–64].

Vancomycin-dependent (VD) bacteria were first reported in 1994, consisting of *Enterococcus faecium* from two patients of separated hospitals in the United Kingdom and an *Enterococcus faecalis* strain from a urine sample of a patient from surgical intensive care. In all cases, patients have had courses of broad spectrum antibiotics including vancomycin [7, 65]. Subsequently, vancomycin dependence has been reported for Enterococci as *E. faecalis* [10, 66–68], *E. faecium* [67, 69–85] and *Enterococcus avium* [86], and also in *S. aureus* [87, 88].

**Fig. 2:**
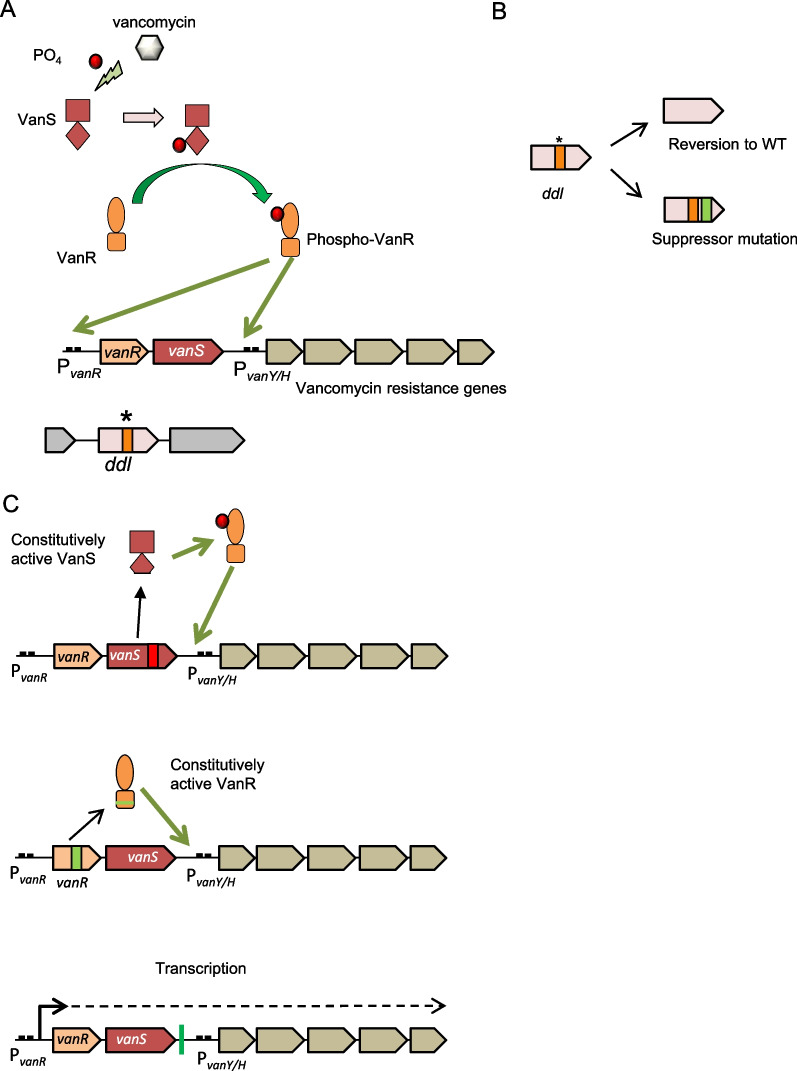
Vancomycin dependence phenotype and its reversion mechanisms.** A** Vancomycin induces the activation by phosphorylation of the histidine kinase-type membranal sensor VanS, which in turns phosphorylates the VanR. Phospho-VanR is then able to activate expression of its own operon and of the genes required for the biosynthesis of the alternative cell wall precursor required to resist vancomycin. Vancomycin-dependent strains display an inactivated *ddl* gene for which they cannot synthesize the original substrate, thus depending on the activation of the whole system provided by vancomycin to thrive. **B** Vancomycin dependence can be reversed by mutations restoring the production of the WT Ddl or by compensatory mutations restoring Ddl activity. **C** Vancomycin dependence can also be reversed by mutations causing antibiotic-independent expression of the vancomycin resistance genes. This can be accomplished by mutations producing constitutively active VanS which activates VanR in the absence of the activation signal (top), mutations leading to constitutively active VanR (middle) or mutations (indicated in green) eliminating an RNA terminator structure downstream the *vanRS* operon, allowing the leaky transcription of resistance genes from the upstream promoter (bottom)

Among all cases of bacterial antibiotic dependence, the dependence to vancomycin is the best understood at the molecular level. Despite the acquisition of the *van* genes conferring the ability to produce the alternative depsipeptides for peptidoglycan synthesis, regular vancomycin-resistant bacteria are still able to synthesize the original _D_-Ala-_D_-Ala precursor. Contrariwise, in most cases studied to date, VD strains are unable to synthesize native _D_-Ala-_D_-Ala (and therefore cell wall) as they harbor inactivating mutations in the *ddl* gene, coding for the _D_-Alanyl–_D_-Alanine ligase. Hence, these strains rely on vancomycin for the induction of the Van genes through the VanRS system for the synthesis of the alternative peptidoglycan precursors, which in this mutant background become essential (Fig. 2A). The fact that exogenous supplementation of _D_-Ala-_D_-Ala may rescue growth in these strains confirms this [7, 72, 80]. Different types of *ddl* inactivating mutations including nonsynonymous mutations [67, 68, 78, 79, 87–90], partial deletions [68] and frameshifts [75, 81, 91] in VD bacteria are reported. In some cases, experimental evidence of the impairment of _D_-Alanine–_D_-Alanine ligase activity of the resultant mutant Ddl protein has been obtained, with reductions in activity ranging from 200 to 1000 fold [79, 87, 90]. Notably, isolates reluctant to grow in the presence of supplemented _D_-Ala-_D_-Ala have also been found, suggesting that currently unknown, alternative vancomycin dependence mechanisms in addition to Ddl inactivation exist [84].

Very frequently, plain vancomycin-resistant revertant derivatives arise from the VD strains. Reported reversion rates from vancomycin dependence to resistance range from 1 in 10^5^ to 1 in 10^6^, with one study reporting as frequent as 1 in 10^3^ [76, 84, 92]. Overall, two different mechanisms of reversion of vancomycin dependence have been documented. The first mechanism implicates the regain of a functional Ddl to relieve the dependence on the alternative peptidoglycan precursor. This could be achieved by mutations reverting the Ddl protein to WT or by compensatory mutations or insertions in the *ddl* gene that restore _D_-Alanine–_D_-Alanine ligase activity [68, 78] (Fig. 2B). The second mechanism involves mutations that allow the expression of the resistance genes in the absence of vancomycin. In the characterized strains, this may be achieved in different ways. First, mutations that render the VanSR system constitutively active have been found. In VanS, amino acid substitutions close to autophosphorylation or histidine kinase domains conserved in this protein, or a 7 amino acid duplication seem to generate this effect in *E. faecalis* strains [68, 78]. Meanwhile, an amino acid substitution seems to generate a constitutively active VanR protein in *S. aureus* [88] (Fig. 2C). Another mechanism of reversion has been described in a VanB-type resistant *E. faecium*. In this strain, the *vanRS*_*B*_ genes are right upstream of the *vanYWHBX* operon. At the end of the *vanRS*_*B*_ operon there is a transcription terminator sequence. A mutation that likely prevents the formation of the transcription terminator structure allows the transcription to proceed towards the downstream *vanYWHBX* genes [79] from the upstream *vanRS*_*B*_ promoter. In such VanB-type cassettes, while the transcription of the promoter of *vanY* is totally dependent on the binding of phosphorylated VanR, a leaky activation of the *vanRS*_*B*_ promoter in the absence of activated VanR exists [93]. Thus, this basal level of expression allows the synthesis of the peptidoglycan precursors even in the absence of the antibiotic when the *vanRS*_*B*_ terminator is inactivated by mutations. Overall, vancomycin dependent strains seem to become dependent on the alternative pathway (the Van resistance cassette) they acquired as a target bypass mechanism to resist vancomycin, because of genetic mutations inactivating their original cell wall biosynthetic pathway. Vancomycin is required as an inductor of the expression of this alternative, now essential pathway. Revertant strains accumulate genetic mutations restoring the activity of the native peptidoglycan biosynthesis or render the Van pathway independent of vancomycin for its expression.

## Bacterial dependence to linezolid

**Fig. 3:**
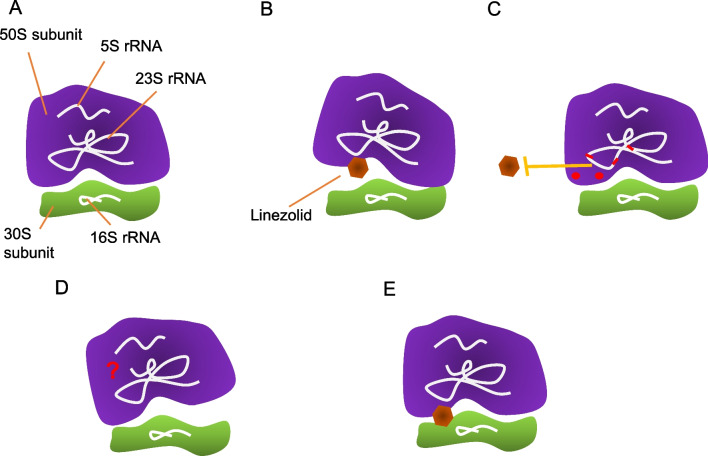
Dependence to linezolid. **A** Depiction of the general components of a normal bacterial ribosome. **B** The mechanism of action of linezolid involves ribosome obstruction by interacting with the 50S and the cognate 23S rRNA. **C** Linezolid resistance mostly involves ribosome modifications both close and distal to the binding site such as 23S rRNA mutations or methylation, or mutations in proteins components of the 50S that preclude linezolid binding. **D** Dependence is setted by the development of linezolid-dependent ribosomes. These ribosomes possess some of the resistance mutations but possibly also undefined mutations or coexist with mutations in other bacterial components that render ribosomes unable to structure correctly in normal conditions. This impediment is amended by the interaction with linezolid in a matter that has not yet been defined (**E**)

Linezolid is a synthetic molecule considered the first member of the class of oxazolidinone antibiotics [94]. This drug is usually employed for the treatment of community- and hospital-acquired pneumonia and skin and soft tissue infections caused by Gram-positive bacteria like methicillin-resistant *S. aureus,* Vancomycin-resistant Enterococci (VRE), penicillin-resistant *S. pneumoniae* and Gram-positive anaerobes [95, 96]. Linezolid obstructs bacterial protein synthesis by binding to the 50S subunit of the prokaryotic ribosome, preventing the formation of the initiation complex at the start of the translation process [97, 98] (Fig. 3A). Studies indicate that linezolid binds the A site in the 50S within the peptidyl transferase center (PTC), interacting with several nucleotides of the cognate 23S rRNA to produce conformational changes that impair peptide bonds formation by blocking the positioning of the tRNA [99–101].

The development of resistance to linezolid in bacteria has been associated with prior, prolonged exposure to the antibiotic. Resistance mechanisms to linezolid include mutations in the 23S rRNA in the PTC, mutations in the L3 and L4 ribosomal proteins, modifications of the 23S rRNA by plasmid-coding methyltransferases (Fig. 3B), and the activity of a plasmid-borne ATP-binding cassette (ABC) transporter [96, 102–107]. The most frequently reported mutation in clinical *Staphylococcus* and *Enterococcus* strains is the G2576T transversion. Nevertheless, mutations in the conserved 23S rRNA nucleotides G2061, A2451, C2452, A2503, T2504, G2505, T2506 and T2585, which directly interact with linezolid but also nucleotides that are not part of the antibiotic binding site like A2062, G2447, A2453, C2499 and T2500 are reported to cause resistance [104, 108].

Linezolid dependence was reported for the first time in five linezolid-resistant *S. epidermidis* isolates from bloodstream infections in a Greek hospital [9]. The growth of these strains was greatly enhanced by relatively high concentrations of linezolid. More recently, two linezolid-dependent (LD) strains of *S. aureus*, both from cystic fibrosis patients who received long antibiotic courses, have been described [22, 109]. The role of linezolid in the activity of the ribosomes of one of the clinical LD *S. epidermidis* isolates was further evaluated. Ribosomes derived from the LD clone have an increased peptidyl-transferase activity in the presence of linezolid. Moreover, without linezolid these ribosomes have an aberrant subunit dissociation profile in sucrose gradient experiments. This suggests that the LD strain developed improved ribosomes, however they are only functional in the presence of this antibiotic because it promotes proper structuration [110]. Strikingly, this resembles the dependence to streptomycin, in the sense that a ribosome-targeting antibiotic seems to induce the emergence of impaired ribosomes whose correct function is restored by the interaction with the antibiotic. Regardless, the relationship of mutations in the 23S and ribosomal proteins or other molecular determinants with the development of linezolid dependence has not been determined. The *S. epidermidis* LD strains harbor specific resistance mutations when compared to linezolid-resistant-only isolates from the same hospital, namely the T2504A/C2534T substitutions in the 23S rRNA and mutations in the L3 protein [9]. Nonetheless, rather than being specifically associated with dependence, this feature could be reminiscent of their clonal origin. Out of the two LD *S. aureus* strains described, one was found to lack any of the known linezolid resistance mutations [109], while the other harbors only the G2576T substitution in all copies of the 23S rRNA [22]. All the mutations described appear in other resistant strains without causing dependence. Thus, no evidence for the generation of dependence by a specific mutation has been obtained. Furthermore, it seems that mutations that lead to linezolid dependence are different from those known to date to confer resistance. In any case, further work is needed to fully disclose the molecular basis and cellular factors involved in the development of dependence to linezolid in bacteria.

As discussed in the introduction, sometimes the acquisition of antibiotic resistance possesses an intrinsic fitness cost. Indeed, acquisition of resistance genes may impose a metabolic burden on the organism and mutation of targets may produce a physiological drawback owed to the relevance of the genes where resistance mutations occurred. Thus, although mutant strains are better fitted to grow in the presence of the antibiotic compared to sensitive strains, they are outcompeted when growing in the absence of the selective pressure [111, 112]. So, it could be speculated that antibiotic dependence comprises a fitness cost associated with the development of resistance. LD *S. epidermidis* strains grow slower than linezolid-sensitive strains in vitro in medium without linezolid. However, linezolid boosts the growth of dependent strains beyond the growth rate of linezolid-sensitive strains without the antibiotic [113]. Although this still needs to be assessed in a set of isogenic linezolid-dependent and sensitive strains, this fact suggests that linezolid dependence, more than survival, confers a further competitive advantage for these strains in patients with prolonged linezolid exposure. Thus, linezolid dependence seems to be more of an example of adaptive evolution over fitness cost traits. However, specifically designed studies are needed in order to properly assess the ecological and evolutionary roles of linezolid (or any antibiotic) dependence. This compelling area of study has been neglected to date.

## Bacterial dependence to colistin

Colistin is a cyclic lipopeptide antibiotic belonging to the polymyxin group, which includes polymyxin E (colistin) and polymyxin B [114, 115]. This drug is the last-resort antibiotic used to treat infections caused by multidrug resistant Gram negative bacteria like *Klebsiella pneumoniae, E. coli, P. aeruginosa* and *Acinetobacter baumannii* [116, 117].

Colistin is a cationic antibiotic that exerts its antimicrobial action via direct interaction of its cationic regions with the negatively charged lipid A of lipopolysaccharide (LPS) localized on the outer membrane [118]. This interaction results in destabilization of the LPS followed by disruption of the outer cell membrane, infiltration of intracellular contents and bacterial death [119]. There are several colistin resistance mechanisms. The main one involves the modification of the structure of the lipid A, causing a decrease in its net negative charge hence inhibiting the colistin initial interaction with the bacterial surface [120]. Lipid A modifications are mediated by the *pmrCAB* and *arnBCADTEF* operons that encode proteins responsible for the addition of phosphoethanolamine (PEtN) or 4-amino-4-deoxy-L-arabinose to the structure, respectively [121–123]. Alternatively, bacteria may display mutations that cause the complete loss of the LPS production [124]. Additional resistance mechanisms include the overexpression of outer membrane proteins, the use of efflux pumps such as MexXY/OprM and AcrAB-TolC, and the plasmid-carried *mcr-1* gene which codes for an enzyme able to transfer PEtN to the lipid A moiety [124–127].

Colistin dependence was first identified during population susceptibility studies of an isolate of the *Acinetobacter baumannii*-*Acinetobacter calcoaceticus* complex from a case of calcaneal osteomyelitis previously treated with colistin. The isolate was subcultured in colistin to study heteroresistance and a subpopulation developed dependence [23]. Thereafter, other studies found in vitro development of colistin dependence in *Acinetobacter baumanni* [24, 128–132]. To date, all reported cases correspond exclusively to strains of *A. baumannii* or *Acinetobacter nosocomialis* [133] that developed antibiotic dependence during successive passages with or without colistin after their isolation from patients. Notably, colistin dependence may also be induced by exposure to the human cationic antimicrobial peptide LL-37 [134].

Dependence is acquired by a significant proportion of colistin-susceptible *A. baumannii* strains. In a survey of clinical isolates, up to one third of them developed colistin dependence after exposure to the antibiotic [135]. In another study, 12% of colistin-heteroresistant *A. baumannii* clinical isolates were found to develop colistin dependence following exposure. These colistin-dependent (CD) isolates belonged to different clonal clusters, suggesting that this phenotype arose many times independently in this group [131].

**Fig. 4:**
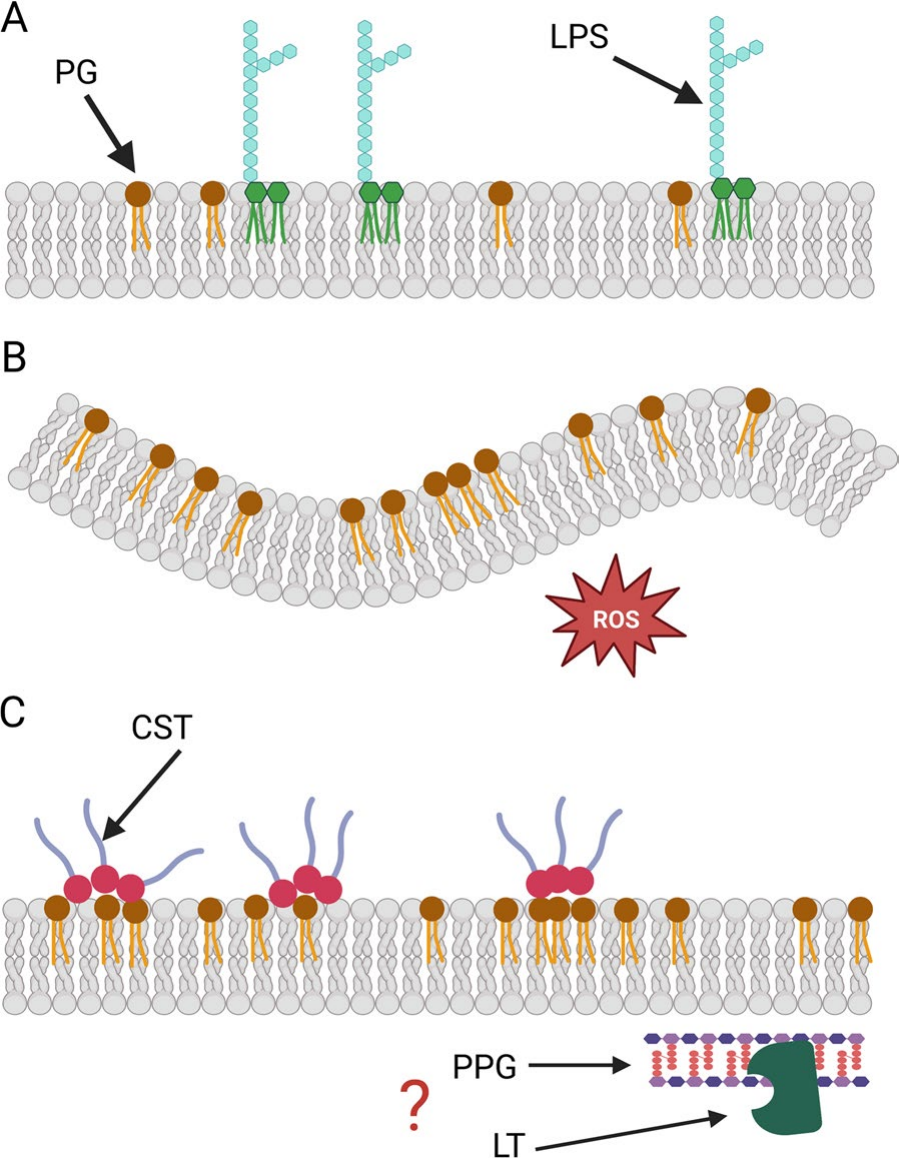
Dependence to colistin. **A** Depiction of a normal *Acinetobacter* outer membrane, with the lipopolysaccharide (LPS) and phosphatidylglycerol (PG) components indicated. **B** Outer membrane of a CD strain. LPS production has been abrogated by genetic mutations. Other mutations are also likely involved in this trait. The membrane has increased PG content and presents a perturbed structure with alterations in the membrane potential. Also, oxidative stress levels are increased. **C** Membrane stabilization by colistin in a CD strain. Colistin binds the PG in the membrane in a localized fashion, increasing the membrane stability. Augmented peptidoglycan turnover by the activity of lytic transglycosylase may also have a role in membrane structure restoration. LPS: lipopolysaccharide, PG: phosphatidylglycerol, ROS: reactive oxygen species, CST: colistin, PPG: peptidoglycan layer, LT: lytic transglycosylase

In 2015, García-Quintanilla and collaborators reported the development of colistin dependence among a subset of colistin-resistant *A. baumannii* isolates. The dependence was exclusively associated with strains that acquired resistance by mutations rendering loss of LPS production. Strains that acquired colistin resistance by LPS modification did not develop dependence [129]. Interruption of the *lpxA*, *lpxC* or *lpxD* genes by insertion sequences (IS) (e.g. IS*Aba1*, ISAjo2, ISAba13 or IS1595), causing the abrogation of lipid A biosynthesis and therefore the loss of lipo-oligosaccharide (LOS), leading to colistin resistance, are also observed in other CD strains [24, 128, 132, 133]. Nonetheless, although probably involved in the CD phenotype, these mutations are not the sole cause of it. As it has been pointed out before, not all lipid A defective *Acinetobacter* strains are colistin-dependent [24]. Moreover, in *A. nosocomialis,* CD was developed without mutations in the *lpxACD* genes [133]. In addition, other mutations in CD strains concomitant with the LOS gene inactivation have been observed. This include mutations in *mlaD* and *pldA* genes required for proper outer membrane (OM) structure composition [128], and mutations in *mrcA,* coding for the penicillin binding protein A1, *katG*, encoding a catalase, *rpoB* coding for the B subunit of the RNA polymerase and in a gene for a putative signaling protein [24]. Nonetheless, the contribution of these mutations to antibiotic dependence is not clear. In general, CD *Acinetobacter* seems to display a perturbed OM structure leading to altered membrane potential, other surface modifications, oxidative stress and increased sensitivity to other antibiotics [24, 128, 132, 134, 136]. A recent study showed that a lytic transglycosylase enzyme involved in cell-wall degradation and recycling was overexpressed in a CD isolate. This enzyme promotes the survival of this strain probably by helping it to cope with the membrane instability caused by LOS lost by increasing peptidoglycan turnover [136]. Overall, the way colistin promotes growth in CD bacteria has only recently started to be elucidated. In a remarkable study, Zhu and coworkers showed that CD emerges in LPS deficient mutants that distinctively undergo OM remodeling with high phosphatidylglycerol (PG) composition at least partially induced by an increase of oxidative stress (Fig. 4A, B). In the CD strain studied, the oxidative stress was likely engendered by the presence of reactive oxygen species accumulated because of the effect of an underlying elimination of *katG*. In this context, polymyxins are able to bind the enriched PG in the OM in a patchy pattern increasing the membrane stability (Fig. 4C) [24]. Noteworthy, in this study overexpression of lytic transglycosylases was also detected, suggesting a common mechanism to support growth in different CD strains.

In general, colistin dependence seems to emerge in a subset of resistant strains that lose LOS production and acquired accompanying genetic mutations collectively leading to membrane redecoration and instability and cellular stress. In such strains, colistin interaction stabilizes the bacterial membrane. Hence, colistin dependence appears to represent an example of fitness cost derived from antibiotic resistance.

## Clinical implications of antibiotic dependence

Many aspects of the role of antibiotic dependence phenotype during an infection are unclear. Particularly, infections caused by VD, LD or CD strains require a better understanding of the implications of dependence in the developments of infection given the clinical importance of these drugs.

It is known that in some cases VD strains may be developed as a collateral effect of antibiotic treatment against multidrug resistant strains of a different pathogen or in cases of supportive treatment to prevent multiple injury infection. Such dependent strains are not considered primary pathogens and are isolated as result of the microbiological surveillance in these patients. Examples of this include an enterococci isolated from a patient receiving vancomycin to treat a sepsis caused by a coagulase-negative *Staphylococcus*, *E. faecium* from the faces of a patient receiving vancomycin to treat methicillin/oxacillin-resistant *S. aureus* and an *E. faecium* isolated from a female patient on intravenous vancomycin therapy for multiple traumatic injuries [71, 75, 82]. In these cases, the pathogenic potential of the strains is not established [92]. Nonetheless, in other cases VD pathogens have been clearly associated with disease states. Bacteremia [10, 72, 78, 81, 84], intraperitoneal, pleural and urinary tract infections [7, 73, 84], and even deaths caused by refractory sepsis associated with VD enterococci strains have been documented [84]. Moreover, the potential of these strains to cause outbreaks associated with health care attention was demonstrated by an outbreak developed by a strain of VD *E. faecium* in five patients in a bone marrow transplant (BMT) unit. Although in this case, no clinical symptoms could be clearly attributed to VD *Enterococcus* colonization and persistence in the patients [76].

In the case of LD strains, the *Staphylococcus* isolates are undoubtedly pathogenic, whether systemic or opportunistic. Furthermore, one of the studies assessed the contribution of linezolid dependence to the dissemination of *S. epidermidis* linezolid resistant strains in Greek hospitals [137]. Strikingly, a majority of linezolid resistant strains (74%) isolated from 2011 to 2013 were actually LD. Almost all LD strains displayed the same macrorestriction pattern and multilocus sequence type, identifying them as belonging to the sequence type ST22. In a similar study among linezolid resistant strains isolated in German hospitals, also most were found to be LD and to belong to the ST22 [113]. The remaining strains belonged to sequence types of the clonal complex 5 (CC5), in which ST22 is located. Hence, it seems that the CC5 lineage of *S. epidermidis* has a predisposition to develop linezolid dependence, and that this trait is clearly involved in the spread of linezolid resistance. Nonetheless, additional experimental research is needed to understand how this may occur.

While all CD strains studied to date were obtained after in vitro passages of clinical strains, many of them were present as heteroresistant components of isolates cataloged as colistin sensitive [131, 133, 135]. Thus, the role of this trait during infection needs clarification. Strikingly, three studies noted that CD strains derived from antibiotic sensitive isolates further develop into stable colistin resistant strains, suggesting that in this particular case, dependence represents an intermediate state towards resistance [131, 132, 135]. Perhaps the most illustrative fact of the importance of this characteristic is that the tendency to develop colistin dependence in clinical *A. baumannii* isolates associates with treatment failure in patients [135]. However, in one of the few studies assessing the implications of dependence in virulence, a CD strain displayed attenuated virulence in murine colonization model compared to its parental non-dependent strain [24].

How the administration of antibiotics is affecting the development of an infection with an antibiotic-dependent strain and whether antibiotic elimination could prompt infection clearance are still paramount open questions. Clearance of the infection just by antibiotic withdrawn may not occur because of the emergence of spontaneous revertants reported in virtually all cases of dependence and the fact that some patients seem to be colonized by a population of heterodependent bacteria, i.e. both dependent and plain resistant colonies are obtained from the patient’s sample [76, 82]. In general, few studies have addressed these questions for any case of antibiotic dependence. In 2010, Zhong and collaborators reported the case of a tuberculosis patient infected by a rifampicin-dependent bacilli. Here, the treatment with an antibiotic cocktail including rifampicin seemed to worsen the disease. Removal of rifampicin and continuation with other antibiotics in the cocktail cured the patient [18]. Although in this case the individual effect of rifampicin elimination and the effects of the rest of the antibiotics in the eradication of the disease could not be circumscribed, it clearly showed that the documentation of the antibiotic dependence was important for the proper design of the regime of drugs. In the case of a VD *Enterococcus* isolated from a urinary tract infection, the vancomycin treatment was substituted by imipenem because of the development of bacteremia and sepsis with a VRE. This cleared the infection both from blood and urine [7]. Thus, in this case it is also not possible to know the specific effect of removing the vancomycin therapy in the resolution of the VRE infection. In a different example, a VD *Enterococcus* causing bacteremia developed in a patient treated with an antibiotic cocktail that included amikacin, imipenem and vancomycin. The enterococci was not further isolated from blood and infection signs disappeared after completion of the treatment [72], which suggests that the antibiotics accompanying vancomycin in the cocktail were effective. Also, due to the development of the VD *Enterococcus* outbreak in a BMT unit described in a previous section, the policy to apply a prophylactic vancomycin treatment to BMT recipients was reviewed and the use of vancomycin was further reserved for serious infections [76]. The effect of linezolid in the infectious outcome when LD strains are involved is to date completely unstudied. Currently, linezolid dependence characterization has only been performed in vitro on standard laboratory media. Research on animal models, at least, is required to assess the effect of linezolid administration/elimination on the virulence of LD strains. As with the case of vancomycin, reversion of LD to resistance only and vice versa seems to be common in *S. epidermidis* [118] and spontaneous generation of plain resistant and sensitive strains derived from a LD *S. aureus* is documented. Thus, it seems that the outcome of infection would not be easily inferred for cases of LD strains and this is one of the more urgent areas of research in antibiotic dependence. Even less is known in this matter regarding CD. Recently, it was shown that a CD *A. baumannii* strain was able to colonize mice and to resist a colistin treatment, in spite of its attenuated virulence [24]. Nonetheless the effect of colistin over CD bacteria during human infections is to date completely undocumented.

Another important remaining issue concerns the pathogen detection practice in the clinical laboratory. Antibiotic-dependent strains have been described as “invisible pathogens” [24] because they pose a challenge for detection by conventional laboratory practices. VD enterococci were only detected by the high antibiotic concentration of the original urine sample or by the routine use of antibiotic discs in the primary isolation plates [7, 65]. LD staphylococci were detected by using long incubation times [22]. Likewise, it has been recently reported that CD *A. baumannii* have a significatively higher incubation time to be detected by hemoculture than the parental colistin-sensitive strains [131]. Thus, standard incubation times and the use of antibiotic-free media in the primary isolation protocols may mask the incidence of dependent bacteria. However, to date it is difficult to estimate the possible contribution of this trait to the underdetection of pathogens. On the basis of the proportion of antibiotic-dependent population of pathogens when ascertained, it may be significant. Certainly, awareness must be raised to include standard laboratory practices allowing the detection of antibiotic-dependent strains. From this, it can be obtained real data regarding the epidemiological importance and complete clinical features of these traits. This information is critical to develop correct treatments.

## Concluding remarks

In light of the current antimicrobial resistance crisis, all of the effects of antibiotics on the physiology of pathogens need to be ascertained. Antibiotic dependence is a trait for which the ecological and evolutionary implications are not always systematically studied. From the information available, it emerges that the basis for dependence relies on specific antibiotics mechanisms of action. For instance, for streptomycin and linezolid, the basis for dependence is at ribosomes function, which are their primary action targets. Similarly, in both colistin sensitivity and dependence membrane structuration processes are involved. While the genetic basis for the dependence are clearly established in some instances (e.g. streptomycin and vancomycin) the association of mutations with other dependencies are not clear. In such cases, a phenotypic-based dependence process cannot be discarded.

The studies reviewed hint into a significant role of dependence in the development and spread of resistance. Likely, pathogen underdetection is also occurring due to this trait. Moreover, in some documented cases, assessing dependence is an important piece of information in order to design proper therapies. All of these are key features to integrate into protocols to withstand multidrug resistance. Moreover, there is a profound knowledge gap in the relationship between dependence and other non canonical bacterial responses to antibiotics, such as heteroresistance, tolerance provided by persisters cells, viable but nonculturable states or heterogeneous antibiotic accumulation. The integral study of these traits through specifically designed research will provide a more accurate landscape of the effects induced by antibiotics and their implications in virulence, epidemiology, diagnostics, and therapy efficacy.

## Data Availability

All data generated or analyzed during this study are included in this published article.

## Abbreviations

WHO: World Health Organization
VRE: Vancomycin Resistant Enterococci
TCS: Two-component system
VD: Vancomycin-dependent
WT: Wild type
PTC: Peptidyl transferase center
MIC: Minimal inhibitory concentration
LD: Linezolid-dependent
LOS: Lipo-oligosaccharide
PG: Phosphatidylglycerol
LPS: Lipopolysaccharide
PEtN: Phosphoethanolamine
CD: Colistin-dependent
IS: Insertion sequences
BMT: Bone marrow transplant
